# ST-segment resolution as a marker for severe myocardial fibrosis in ST-segment elevation myocardial infarction

**DOI:** 10.1186/s12872-021-02269-y

**Published:** 2021-09-21

**Authors:** Qian Dong, Xuesong Wen, Guanglei Chang, Rui Xia, Sihang Wang, Yunjing Yang, Yi Tao, Dongying Zhang, Shu Qin

**Affiliations:** 1 Department of Cardiology, The First Affiliated Hospital of Chongqing Medical University, Yuzhong, Chongqing, China; 2 Chongqing Medical University, Yuzhong, Chongqing, China; 3 Department of Radiology, The First Affiliated Hospital of Chongqing Medical University, Yuzhong, Chongqing, China

**Keywords:** ST-segment resolution, ST-elevation myocardial infarction, Percutaneous coronary intervention, Myocardial scar

## Abstract

**Objective:**

To investigate the relationship between ST-segment resolution (STR) and myocardial scar thickness after percutaneous coronary intervention (PCI) in patients with ST-segment elevation myocardial infarction (STEMI).

**Methods:**

Forty-two STEMI patients with single-branch coronary artery stenosis or occlusion were enrolled. ST-segment elevations were measured at emergency admission and at 24 h after PCI. Late gadolinium-enhanced cardiac magnetic resonance imaging (CMR-LGE) was performed 7 days after PCI to evaluate myocardial scars. Statistical analyses were performed to assess the utility of STR to predict the development of transmural (> 75%) or non-transmural (< 75%) myocardial scars, according to previous study.

**Results:**

The sensitivity and specificity of STR for predicting transmural scars were 96% and 88%, respectively, at an STR cut-off value of 40.15%. The area under the curve was 0.925. Multivariate logistic proportional hazards regression analysis disclosed that patients with STR < 40.15% had a 170.90-fold higher probability of developing transmural scars compared with patients with STR ≥ 40.15%. Pearson correlation and linear regression analyses showed STR percentage was significantly associated with myocardial scar thickness and size.

**Conclusion:**

STR < 40.15% at 24 h after PCI may provide meaningful diagnostic information regarding the extent of myocardial scarification in STEMI patients.

**Supplementary Information:**

The online version contains supplementary material available at 10.1186/s12872-021-02269-y.

## Introduction

Transmural myocardial scars of the left ventricle that complicate ST-segment elevation myocardial infarction (STEMI) predispose to heart failure and cardiac death. Therefore, early identification and analysis of myocardial scars are particularly important [1]. The size and tissue heterogeneity of healing scars identified by late gadolinium-enhanced cardiac magnetic resonance imaging (CMR-LGE) are independent predictors of arrhythmia and sudden cardiac death [2–7]. CMR-LGE is the gold standard for the diagnosis of myocardial scar and assessment of myocardial salvage [8–11], but is expensive, time-consuming, unfit for wide population studies, and generally contraindicated in patients with cardiac implants. These disadvantages have restricted its clinical application.

Delayed ST-segment resolution (STR) is prevalent during major adverse cardiovascular events, and is predictive of arrhythmia, heart failure, and 30-day mortality [12]. We hypothesized that analysis of STR could represent a convenient, widely accessible, and inexpensive diagnostic method for patients who cannot tolerate CMR-LGE. Consequently, the aim of our study was to establish whether poor STR, as well as CMR-LGE findings, can detect myocardial scarification in the early post-infarction period in STEMI patients.

## Methods

### Study design and population

Forty-two consecutive patients with STEMI treated with coronary angiography and percutaneous coronary intervention (PCI) within 12 h of the onset of pain were enrolled between March 2017 and October 2017. Inclusion criteria were (1) single-branch coronary artery stenosis or occlusion, and (2) restoration of coronary perfusion to TIMI flow grade 3 after PCI. Exclusion criteria were a prior history of the acute coronary syndrome; coronary revascularization; severe chronic kidney disease; intracardiac pacing leads or other implants precluding CMR-LGE; hemodynamic instability; or known claustrophobia. The study was performed at the First Affiliated Hospital of Chongqing Medical University, China. Demographic and clinical characteristics including ECG STR 24 h after PCI were recorded. CMR-LGE was performed 7 days after PCI. This study was conducted in accordance with the Declaration of Helsinki. The research protocol was approved by the locally appointed Ethics Committee, and written informed consent was obtained from all study participants.

### CMR-LGE protocols

Patients were examined in the supine position using a 1.5-T imaging unit (Signa Infinity Twinspeed, General Electric Healthcare, USA) equipped with master gradients (30 mT/m peak gradients; 150 mT/m/ms slew rate) and a 5-element cardiac phased-array receiver coil. Images were obtained using electrocardiographic gating and expiratory breath holds. A dose of 0.2 mmol/kg of body weight of gadopentetate dimeglumine (Magnevist; Bayer Schering Health Care, Cambridge, UK) was administered intravenously at a rate of 5 ml/s with a power injector. Ten minutes after contrast agent injection, a Look-Locker sequence was performed to obtain the most appropriate inversion time to nullify the signal intensity of normal myocardium. The left ventricular short-axis imaging layer was 8 mm thick and 0 mm apart. The left ventricular 2-chamber and 4-chamber scanning imaging layers were 5 mm thick and 0 mm apart. This was immediately followed by the acquisition of LGE images, with an inversion recovery prepared T1-weighted gradient-echo sequence (4.9/1.9; flip angle, 15 degrees; turbo field-echo factor, 30; spatial resolution, 1.35 × 1.35 × 10 mm). Late gadolinium enhancement was interpreted as present or absent by the consensus of two CMR-trained physicians, and was considered present only if confirmed on both short-axis and matching long-axis myocardial locations.

First, we marked the segments of the myocardial scar with the bull’s eye segmental comparison (17-segment model) and compared them with the results of coronary angiography. Second, we searched for the thickest myocardial scar layer-by-layer on the short axis imaging and calculated the percentage of the thickness of the myocardial scar, which was defined as a transmural myocardial scar when the percentage was > 75%, according to previous study [13]. Beek et al. demonstrated that the transmural extent is relevant in predicting myocardial functional recovery: The likelihood of complete functional recovery of segments without LGE was 3.8, 11.1, and 50 times higher than that of segments with 26–50%, 51–75%, and > 75% LGE, respectively (*P* < 0.001) [13]. Finally, we recorded the area of myocardial scar and calculated the percentage of myocardial scar volume by segment software.

### ECG protocols

Standard 18-lead ECGs were obtained at emergency admission and 24 h after coronary angiography and PCI. The TP segment was used as the isoelectric line in the ST-segment measurement. The ST segment was measured 20 ms after the J point. The summed ST-segment elevation was measured by adding the ST amplitude in all leads with ST-elevation at emergency admission and 24 h after PCI [14–16]. STR percentage was calculated as the initial sum of ST-segment elevation minus the sum of ST-segment elevation on the second ECG, divided by the initial sum of ST-segment elevation.

### Statistical analysis

Basic descriptive statistics were used. CMR-LGE location data were described on a patient-by-patient basis. Statistical analyses were performed to assess the clinical utility of using of STR to predict myocardial scarification. Myocardial scars were assessed using two measures: (1) scar thickness and (2) scar size. Receiver operating characteristic (ROC) curve analysis, logistic regression analysis, and multivariate logistic proportional-hazards regression estimated transmural myocardial scar. Pearson correlation and linear regression analyses were used to investigate the coefficients of STR percentage with myocardial scar thickness and size. Myocardial scar thickness and size difference between two ST-segment resolution groups were analyzed by *t*-test. All statistical analyses were performed using SPSS v.22.0 (IBM, Armonk, NY, USA). *P* value < 0.05 was considered statistically significant.

## Results

### Location, size, thickness of the myocardial scar determined by CMR-LGE

Myocardial scars were diagnosed in 41 of 42 STEMI patients (96.7%) by using CMR-LGE. A patient-by-patient visual analysis of scar tissue location in the STEMI group, with bull’s eye segmental comparison of CMR-LGE findings is shown in Additional file 1: Fig. S1. In all patients, the anatomic locations of scars defined by CMR-LGE corresponded to the distributions of the culprit vessels treated with primary angioplasty. For example, in a patient with angiographically proven left anterior descending coronary artery occlusion, CMR-LGE indicated scarification of the basal and middle segments of the left anterior ventricular wall. In another patient with right coronary artery occlusion, CMR-LGE disclosed a scar that involved the entire inferior wall of the left ventricle and the middle and apical segments of the posterior interventricular septum (Additional file 1: Fig. S2). However, scar size and thickness were unrelated to the degree of coronary artery occlusion. Stenoses in all culprit arteries exceeded 90%; nonetheless, there were significant inter-patient differences in scar size and thickness (*P* < 0.001).

### Determination of STR cut-off value

**Fig. 1:**
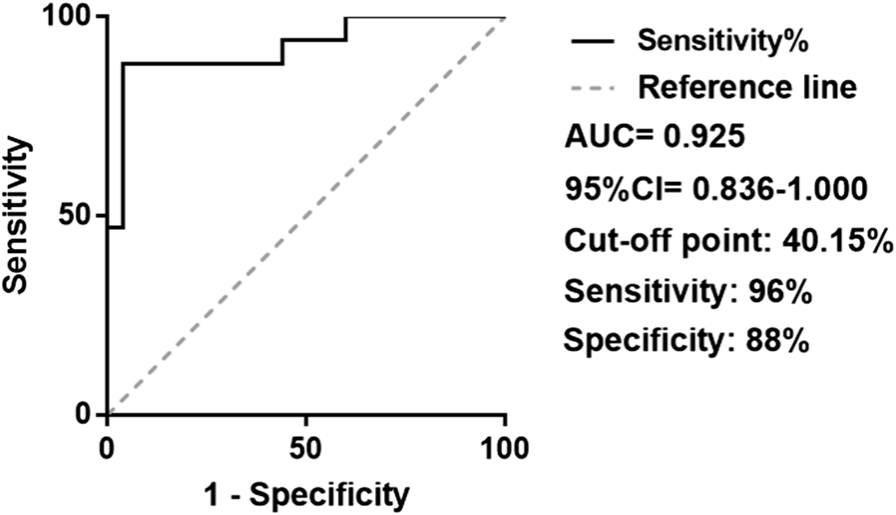
ST-segment resolution with transmural myocardial scar. ROC curve analysis demonstrated the sensitivity and specificity of ST-segment resolution for predicting transmural cardiac scarring after STEMI were 96% and 88%

A transmural scar was defined as a myocardial lesion extending > 75% of the wall thickness. All myocardial scars were classified as either non-transmural (0–75%) or transmural (76–100%) according to CMR-LGE results. The relationship of the ST-segment resolution percentage to transmural scarification was identified by the ROC curve. The ROC curve analysis demonstrated a sensitivity of 96% and a specificity of 88% to predict transmural myocardial scarification following STEMI at an STR cut-off value of 40.15%. The area under the curve was 0.925 (Fig. 1).

The cut-off of ST-segment resolution of 40.15%. Area under curve (AUC) = 0.925.

### Patient characteristics of STR groups

Patients were divided into two groups: STR < 40.15% (n = 16) and STR ≥ 40.15% (n = 26). Compared with patients with STR ≥ 40.15%, patients with STR < 40.15% had significantly longer pain-to-balloon time; higher aspartate aminotransferase (AST), peak troponin-I, and brain natriuretic peptide (BNP) levels; lower left ventricular ejection fraction (LVEF); and a higher prevalence of diuretic therapy. Other characteristics such as age; gender; body mass index; histories of smoking, hypertension, and diabetes; hyperlipidemia; culprit artery; leukocyte, erythrocyte, and platelet counts; hemoglobin, hemoglobin A1(c), alanine aminotransferase (ALT), and creatinine levels; left ventricular diastolic diameter; the use of therapeutic drugs other than diuretics; and coronary care unit residence time were similar between the two groups (Table 1).

**Table 1:** Baseline patient characteristics of different degrees of ST-segment resolution

Variables	All (n = 42)	ST-segment resolution ≥ 40.15%	ST-segment resolution < 40.15%	*P*
		(n = 26)	(n = 16)	
Male/female (n)	30/12	20/6	10/6	0.483
Age (years)	62.0 (54.5–70.0)	60.5 (52.8–70.3)	63.5 (59.5–69.5)	0.534
BMI (Kg/m^2^)	24.2 (23.0–25.6)	24.3 (23.4–25.8)	24.2 (21.9–24.7)	0.378
Smoking history (No, %)	27 (64.3%)	17 (65.4%)	10 (62.5%)	0.850
HP history (no, %)	27 (64.3%)	14 (53.8%)	13 (81.2%)	0.072
DM history (no, %)	16 (38.1%)	12 (46.2%)	4 (25.0%)	0.170
Hyperlipidemia (no, %)	21 (50.0%)	13 (50.0%)	8 (8.0%)	1.000
Pain to balloon time (h)	5.79 (3.67–7.60)	4.33 (3.50–6.23)	7.04 (5.81–8.40) *	0.001
Killip classification (no, %)				0.757
Killip I	29 (74.4%)	19 (79.2%)	10 (66.67%)	
Killip II	10 (25.6%)	5 (20.8%)	5 (33.33%)	
Number of DES (no, %)				0.142
One	34 (87.2%)	22 (95.7%)	12 (75.0%)	
Two	3 (7.7%)	1 (4.3%)	2 (12.5%)	
Three	2 (5.1%)	0 (0.0%)	2 (12.5%)	
Culprit artery, (No, %)				0.171
LAD	18 (42.9%)	8 (30.8%)	10 (62.5%)	
LCC	11 (26.2%)	8 (30.8%)	3 (18.8%)	
RCA	13 (31.0%)	10 (38.5%)	3 (18.8%)	
WBC count (10^9^/L)	9.85 (8.28–12.06)	10.09 (8.28–12.06)	9.72 (8.03–14.12)	0.875
RBC count (10^12^/L)	4.44 (3.91–4.82)	4.44 (4.14–4.86)	4.45 (3.71–4.72)	0.449
Hb (g/L)	139.0 (125.0–151.0)	139.0 (128.0–151.0)	135.5 (113.3–150.8)	0.449
PLT (10^9^/L)	198.0 (163.0–238.0)	195.0 (160.0–213.0)	212.5 (175.0–282.3)	0.204
HbA1c (%)	6.0 (5.7–7.2)	6.1 (5.7–8.2)	5.9 (5.7–6.4)	0.188
ALT (U/L)	42.0 (35.0–56.0)	41.0 (35.0–56.0)	46.0 (36.0–53.5)	0.689
AST (U/L)	126.0 (52.0–209.0)	75.0 (35.0–153.0)	185.5 (101.1–237.3)	0.015
Cr (umol/L)	73.0 (60.0–81.0)	73.0 (59.0–81.0)	71.5 (60.3–83.3)	0.954
BNP (pg/ml)	57.9 (7.9–245.0)	11.7 (5.0–62.7)	227.5 (80.0–381.2) *	< 0.001
Peak troponin-T (ng/ml)	11.6 (6.2–19.3)	7.0 (3.8–14.0)	18.6 (12.2–24.9) *	< 0.001
LVIDd (mm)	49.0 ± 3.3	49.0 ± 3.3	49.5 ± 3.2	0.298
LVEF (%)	53.5 (48.0–58.25)	57.0 (53.5–61.0)	47.5 (44.3–50.0) *	< 0.001
Scar thickness	70.0 (55.0–80.0)	59.0 (50.0–70.0)	81.0 (80.0–85.0) *	< 0.001
Transmural myocardial scar (no, %)	17 (40.5%)	2 (7.7%)	15 (93.8%)*	< 0.001
Scar size	17.35 (12.23–20.30)	12.90 (11.13–19.40)	20.00 (18.13–23.63) *	0.001
IIa/IIIb inhibitor (no, %)	17 (43.6%)	11 (47.8%)	6 (37.5%)	0.522
Aspirin (no, %)	39 (100.0%)	23 (100.0%)	16 (100.0%)	1.000
Ticagrelor/clopidogrel (no, %)	39 (100.0%)	23 (100.0%)	16 (100.0%)	1.000
Statins (no, %)	39 (100.0%)	23 (100.0%)	16 (100.0%)	1.000
β-blocker (no, %)	31 (79.5%)	17 (73.9%)	14 (87.5%)	0.432
ACEI/ARB	24 (61.5%)	14 (60.9%)	10 (62.5%)	0.918
Nitrates (no, %)	38 (97.4%)	22 (95.7%)	16 (100.0%)	1.000
Diuretics (no, %)	16 (41.0%)	6 (26.1%)	10 (62.5%)	0.023
CCU (hours)	48.0 (39.0–62.0)	47.0 (38.0–58.0)	52.5 (44.0–82.8)	0.123

Data are data are presented as mean ± SD, median (interquartile ranges), or number (%)

*STEMI* ST-segment elevation myocardial infarction, *BMI* body mass index, *HP* hypertension, *DM* diabetes mellitus, *DES* drug eluting stents, *LAD* left anterior descending coronary artery, *LCC* left circumflex coronary artery, *RCA* right coronary artery, *LVIDD* left ventricular diastolic diameter, *LVEF* left ventricular ejection fraction, *ACEI* angiotensin converting enzyme inhibitors, *ARB* angiotensin receptor blocker, *CCU* coronary heart disease care unit

*P < 0.05 comparing with ST-segment resolution ≥ 40% group

### Diagnostic value of STR < 40.15% for transmural myocardial scar

In the logistic regression analysis, LVEF (OR = 0.520, 95% CI 0.341–0.792), and STR < 40.15% (OR = 15.0, 95% CI 1.981–113.556) were significant risk factors for transmural scars (Table 2).

**Table 2:** Effects of various variables on transmural myocardial scar in logistic regression analysis

Characteristics	OR (95%CI)	*P*
Male, yes versus no	0.500 (0.234–1.068)	0.074
Age, per 1 years	0.994 (0.984–1.004)	0.238
BMI, per 1 kg/m^2^	0.984 (0.959–1.009)	0.206
Smoking history, yes versus no	0.588 (0.269–1.285)	0.183
HP history, yes versus no	1.077 (0.506–2.291)	0.847
DM history, yes versus no	0.333 (0.108–1.034)	0.057
Hyperlipidemia, yes versus no	0.615 (0.255–1.485)	0.280
Pain to balloon time, per 1 min	1.018 (0.928–1.118)	0.702
Killip classification, II versus I	1.500 (0.423–5.315)	0.530
Number of DES, two & three versus one	4.000 (0.447–35.788)	0.215
Culprit artery: RCD	1.00	
Culprit artery: LAD	0.571 (0.167–1.952)	0.372
Culprit artery: LCC	0.444 (0.137–1.443)	0.177
WBC count, per 1 × 10^9^/L	0.971 (0.917–1.028)	0.317
RBC count, per 1 × 10^12^/L	0.915 (0.792–1.058)	0.229
Hb, per 1 × g/L	0.997 (0.993–1.002)	0.241
PLT, per 1 × 10^9^/L	0.999 (0.996–1.002)	0.567
HbA1c, per 1%	0.936 (0.852–1.029)	0.172
ALT, per 1U/L	0.995 (0.984–1.007)	0.442
AST, per 1U/L	1.001 (0.998–1.004)	0.561
Cr, per 1 umol/L	0.995 (0.987–1.004)	0.283
BNP, per 1 pg/ml	1.003 (0.999–1.008)	0.120
Peak troponin-T, per 1 ng/ml	1.014 (0.975–1.054)	0.500
LVIDd, per 1 mm	0.993 (0.980–1.005)	0.255
LVEF, per 1%	0.520 (0.341–0.792)	0.002
ST-segment < 40.15%, yes versus no	15.0 (1.981–113.556)	0.009
Scar thickness	1.000 (0.991–1.009)	0.975
Scar size	0.993 (0.960–1.027)	0.685
IIa/IIIb inhibitor, yes versus no	0.545 (0.202–1.475)	0.232
β-blocker, yes versus no	0.824 (0.406–1.671)	0.591
ACEI/ARB, yes versus no	0.846 (0.379–1.889)	0.683
Nitrates, yes versus no	0.727 (0.382–1.385)	0.332
Diuretics, yes versus no	2.200 (0.764–6.332)	0.144
CCU, per 1 h	1.000 (0.989–1.011)	0.987

*STEMI* ST-segment elevation myocardial infarction, *BMI* body mass index, *HP* hypertension, *DM* diabetes mellitus, *DES* drug eluting stents, *LAD* left anterior descending coronary artery, *LCC* left circumflex coronary artery, *RCA* right coronary artery, *WBC* white blood cell, *RBC* red blood cell, Hb haemoglobin, *PLT* platelet count, *HbA1c* hemoglobin A1c, *ALT* alanine aminotransferase, *AST* aspartate transaminase, *Cr* creatinine, *BNP* brain natriuretic peptide, *LVIDD* left ventricular diastolic diameter, *LVEF* left ventricular ejection fraction, *ACEI* angiotensin converting enzyme inhibitors, *ARB* angiotensin receptor blocker, *CCU* coronary heart disease care unit

Multivariate logistic proportional hazards regression analyses were used to evaluate the independent predictive value of STR < 40.15%. After adjusting for BNP, Peak Troponin-I, AST, the OR of STR < 40.15% for transmural scar was 170.90 (95% CI 2.26–12,953.74, *P* = 0.020). STR < 40.15% showed significance for predicting transmural scar (Table 3).

**Table 3:** Results of multivariate logistic proportional-hazards regression analyzing the effect of baseline variables on transmural myocardial scar

Model	OR (95%CI)	*P*
Not adjusted ST-segment resolution < 40.15%, yes versus no	15.0 (1.98–113.56)	0.009
Model		
ST-segment resolution < 40.15%, yes versus no	170.90 (2.26–12,953.74)	0.020
BNP, per 1 pg/ml	1.01 (0.99–1.02)	0.313
Peak troponin-I, per 1 ng/ml	0.95 (0.75–1.21)	0.695
AST, per 1U/L	1.00 (0.99–1.02)	0.615

*STEMI* ST-segment elevation myocardial infarction, *BNP* brain natriuretic peptide, *LVEF* left ventricular ejection fraction, *AST* aspartate transaminase

### Relationship between STR percentage and myocardial scar thickness and size

**Fig. 2:**
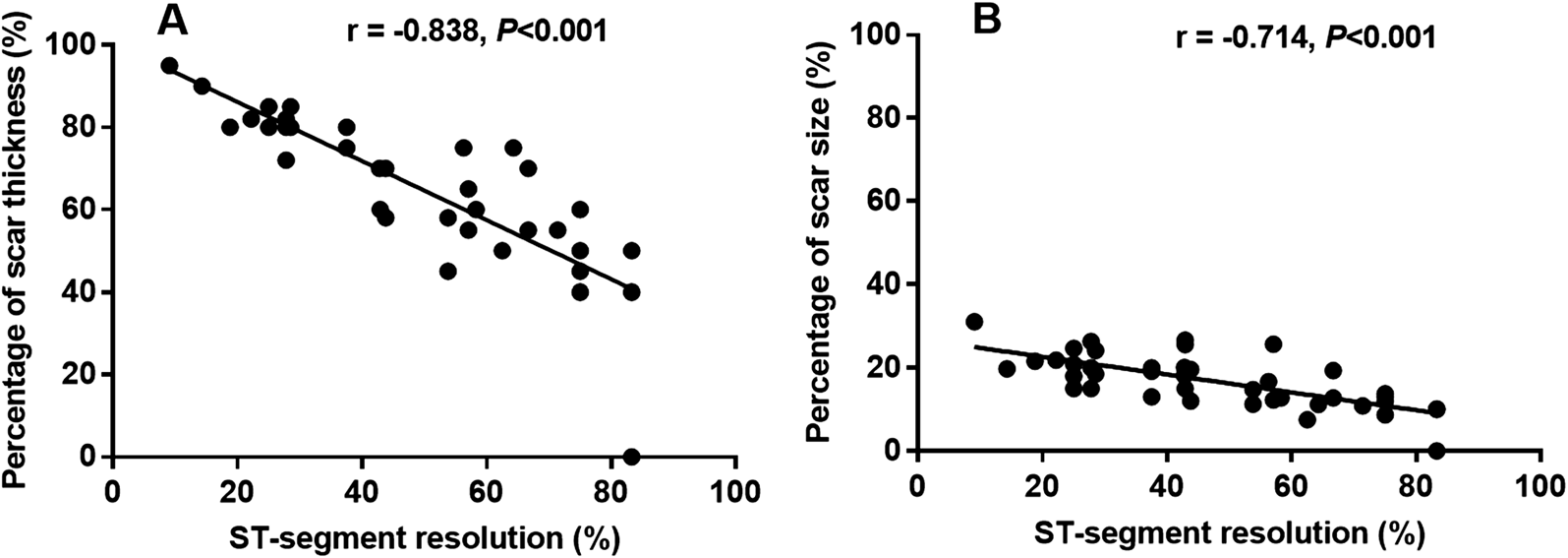
Pearson correlation analysis between ST-segment resolution and myocardial scar thickness or size. **A** Pearson correlation analysis was performed to evaluate ST-segment resolution was negatively correlated with scar thickness. r = − 0.838, *P* < 0.001. **B** Pearson correlation analysis was performed to evaluate ST-segment resolution was negatively correlated with scar size. r = − 0.714, *P* < 0.001

**Fig. 3:**
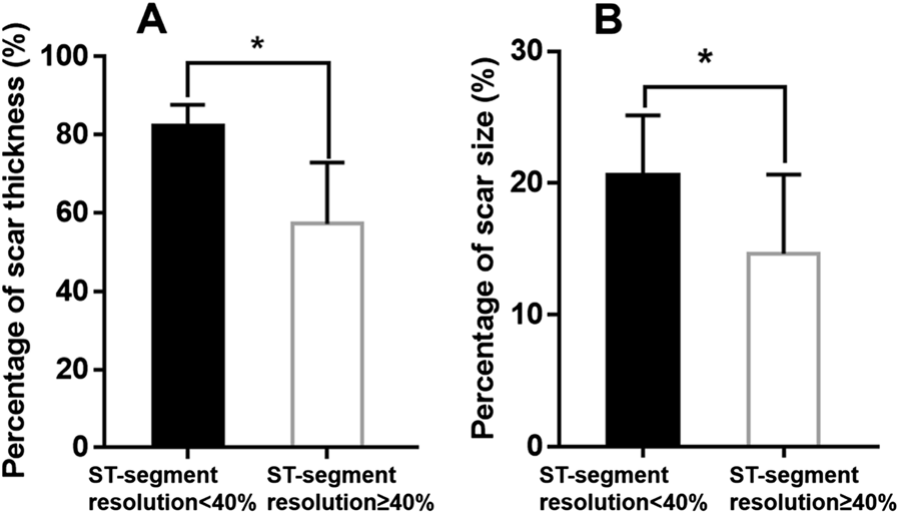
Myocardial scar thickness and size difference between two ST-segment resolution groups. **A** Myocardial scar thickness was statistically significant in patients with ST-segment resolution < 40.15% and in patients with ST-segment resolution ≥ 40.15% (t-Test). Data are reported as mean ± SD. *P < 0.001. **B** Myocardial scar size was statistically significant in patients with ST-segment resolution < 40.15% and in patients with ST-segment resolution ≥ 40.15% (t-Test). Data are reported as mean ± SD. *P < 0.001

Pearson correlation analysis demonstrated negative correlations between STR percentage and both scar thickness (r = − 0.838, *P* < 0.001) and size (r = − 0.714, *P* < 0.001) (Fig. 2). Linear regression analysis with STR (%) as the independent variable and myocardial scar thickness and size as dependent variables were performed. For every 1% decrease in STR, the myocardial scar thickness and size decreases by 0.718% (95% CI − 0.867 ~ − 0.568, *P* < 0.001) and 0.214% (95% CI − 0.281 ~ − 0.147, *P* < 0.001), respectively (Table 4). In addition, compared with patients with STR > 40.15%, patients with STR < 40.15% had significantly thicker and larger scars (Fig. 3).

**Table 4:** Results of linear regression analysis with ST-segment resolution (%) as the independent variable and myocardial scar thickness and size as dependent variables

Dependent variables	R2	B	Std.error	t	*P*	95% CI for B
Myocardial scar thickness	0.702	− 0.718	0.074	− 9.707	< 0.001	(− 0.867, − 0.568)
Myocardial scar size	0.510	− 0.214	0.033	− 6.453	< 0.001	(− 0.281, − 0.147)

## Discussion

Our study showed that ECG, as a convenient and non-invasive technique, can monitor the occurrence of transmural myocardial fibrosis after acute myocardial infarction. In our study, it is possible to detect myocardial scar by CMR-LGE at 7 days after PCI, even when PCI is performed within 12 h of the onset of pain. Many previous studies have focused on the correlation between STR in ECG and poor prognosis in patients with MI, but no cutoff value and clinical markers have been formed. Our study attempted to quantify the predictive value of STR for transmural myocardial scar after STEMI using a small sample.In our study population, there were no statistically significant differences in gender, age, BMI, smoking history, hypertension history, diabetes history, hyperlipidemia history, culprit artery, number of stent implantation, hemogram, HbA1c, ALT, CCU time and the use of drugs including IIa/IIIb inhibitor, Aspirin, Ticagrelor/Clopidogrel, Statins, β-blocker, ACEI/ARB, Nitrates, Diuretics. However, compared with the STR ≥ 40.15% group, the incidence of transmural myocardial fibrosis was higher (*P* < 0.001) and the size of myocardial scar was larger (*P* = 0.001) in the STR < 40.15% group, the difference was statistically significant (Table 1).

Although many researchers believe the association of ST segment resolution and transmural myocardial scar, no one has really verified and quantified it. The association between them may help diagnose myocardial fibrosis simply and quickly in the early clinical stage. Therefore, we use actual cases to verify and quantify the connection between ST segment resolution and transmural myocardial scar. It is significant to quantify STR and apply such a convenient and non-invasive technique to the clinical monitoring of transmural fibrosis in myocardial infarction. Although CMR-LGE is the gold standard for the diagnosis of myocardial fibrosis, patients with cardiac scar often cannot tolerate CMR-LGE and other methods are needed to diagnose transmural scar. STR is a useful predictor of the final infarct size, left ventricular function, and clinical outcome after early reperfusion [17–19]. Nonetheless, the predictive value of STR is still controversial [20, 21]. Rakowski et al. showed that STR < 70% is a marker of large infarct size [21]. However, the transmural depth of an infarction is more consequential than its size. To the best of our knowledge, there are no data to date on the relationship between STR and the transmural thickness of infarcted myocardium. Therefore, our study was focused on defining a specific cut-off value of STR for the diagnosis of transmural scars. We found that the predicted critical value of STR of a transmural myocardial scar after STEMI was 40.15%, with a sensitivity of 96% and a specificity of 88% (Fig. 1). Poor STR was related to thicker and larger scars (Figs. 2, 3). Previous studies have reported relationships between STR and reduced myocardial perfusion and between early STR and myocardial rescue [22]. In addition, STR following PCI and restoration of perfusion to TIMI flow grade 3 was correlated with collateral circulation [23]. STR < 50% may be associated with worse left ventricular function and increased mortality [11, 24]. These results are consistent with our findings, but our study found that patients with STR > 40.15% had thinner and smaller myocardial scar.

STR is significant in monitoring the prognosis and treatment of transmural myocardial fibrosis after myocardial infarction. Transmural myocardial scars are caused by ischemic injury followed by fibrosis of necrotic tissue [1, 25–27]. Poor perfusion, limited myocardial salvage, and microvascular disease promote scarification. The severity and localization of ventricular wall injuries are also influenced by the length of coronary artery stenosis and the degree of collateral circulation [28–30]. The extent of myocardial fibrosis is an important determinant of prognosis. In STEMI, transmural MF usually leads to irreversible ventricular remodeling and heart failure, and is also the pathological basis for arrhythmias, and is generally considered to be associated with sudden cardiac death [31–34]. Nguyen et al. [35] found that the severity of myocardial fibrosis was significantly correlated with the frequency of ventricular arrhythmias (r = 0.83, *P* < 0.01). Our study showed that the STR < 40.15% group had a higher incidence of transmural myocardial fibrosis, and no differences in the incidence of arrhythmias and readmission rate were found between the two groups, which may be related to the small sample size (Additional file 1: Fig. S3). Scientists' understanding of the mechanisms and consequences of cardiac fibrosis has only improved greatly in recent years, with the improvement of non-invasive techniques to better track its development [36]. It is significant to explore the effect of STR on the prognosis of transmural myocardial fibrosis after myocardial infarction in a larger sample and a longer follow-up time.

The physiology of STR is related to the restoration of myocardial perfusion. Following PCI of epicardial coronary arteries, microvascular spasm and embolism may lead to persistent coronary microvascular dysfunction (CMD) and subsequently cause myocardial and especially endocardial ischemia. In the setting of CMD, extracellular potassium ion clearance is decreased, thus prolonging repolarization and delaying STR. Poor STR reflects microvascular and left ventricular dysfunction [20, 37, 38], and is thereby an important biomarker of CMD after PCI in STEMI patients. Inadequate perfusion due to CMD is the proximate cause of transmural myocardial injury; consequently, assessment of the severity of ischemia by monitoring dynamic ST-segment changes is of the utmost importance. In our study, it was found that compared with the group with STR ≥ 40.15%, the pain to balloon time was significantly prolonged (*P* = 0.001), the left ventricular ejection fraction was significantly reduced (*P* < 0.001), and the troponin and BNP were higher (*P* < 0.001) in the group with STR < 40.15%. The delay of opening time of effective coronary blood flow in STR < 40.15% group may be an important reason for the aggravation of myocardial transmural injury and the formation of transmural myocardial fibrosis. Shortening the pain to balloon time may avoid or reduce the occurrence of poor STR, thus affecting the clinical prognosis of patients.

## Limitations

There are several limitations of this study. First, this was a cross-sectional study with a relatively small number of patients. Second, it was difficult to recruit STEMI patients who were willing or able to undergo CMR-LGE 7 days after PCI. Third, because this study was limited to STEMI patients, cross-validation analysis is needed to determine whether delayed STR can be used to predict myocardial scarification in non-STEMI patients. Fourth, to verify the clinical effects of STR, longer clinical follow-up is needed, especially for the monitoring and follow-up of malignant arrhythmias.

## Conclusion

We found that STR correlated with myocardial scar thickness following STEMI. To the best of our knowledge, this is the first study to confirm that STR < 40.15% after PCI can provide important prognostic information regarding myocardial fibrosis in STEMI patients. These results suggest that STR may represent a safe, readily accessible, easily administered, inexpensive diagnostic modality in the management of STEMI patients for whom CMR-LGE is contraindicated.

## Supplementary Information

**Additional file 1.** Eye representation of segments in which visual analysis of CMR-LGE detected scar tissue.

## Data Availability

The datasets used and analyzed during the current study are available from the corresponding author on reasonable request.
