# Supplementary material for: ST-segment resolution as a marker for severe myocardial fibrosis in ST-segment elevation myocardial infarction
Source: BMC Cardiovasc Disord. 2021 Sep 21;21:455. doi: 10.1186/s12872-021-02269-y (PMC8454141; doi:10.1186/s12872-021-02269-y)
Supplement: Supplementary file 1 — Additional file 1. Eye representation of segments in which visual analysis of CMR-LGE detected scar tissue. [file 12872_2021_2269_MOESM1_ESM.pdf]

Supplemental Figure 1.

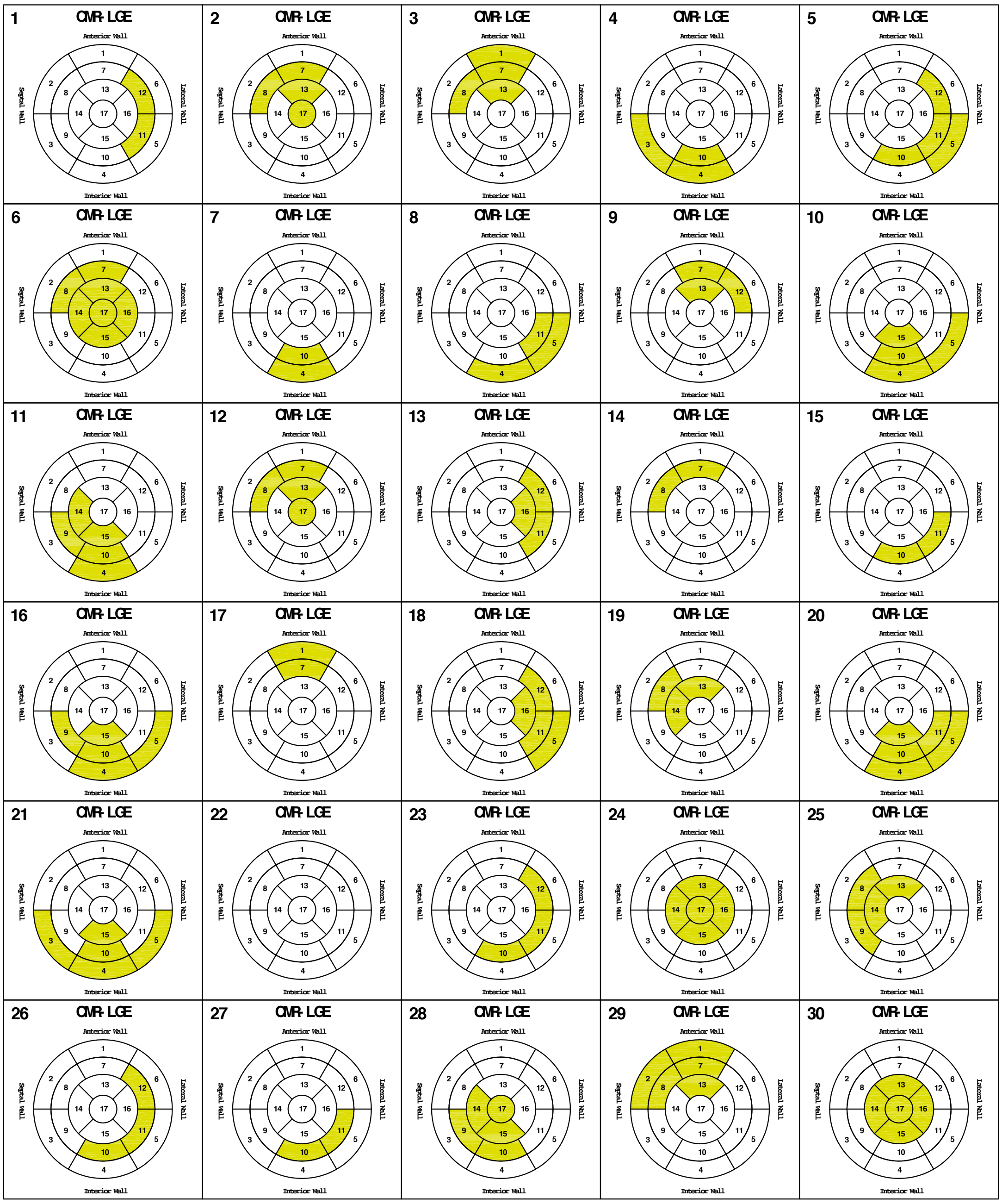

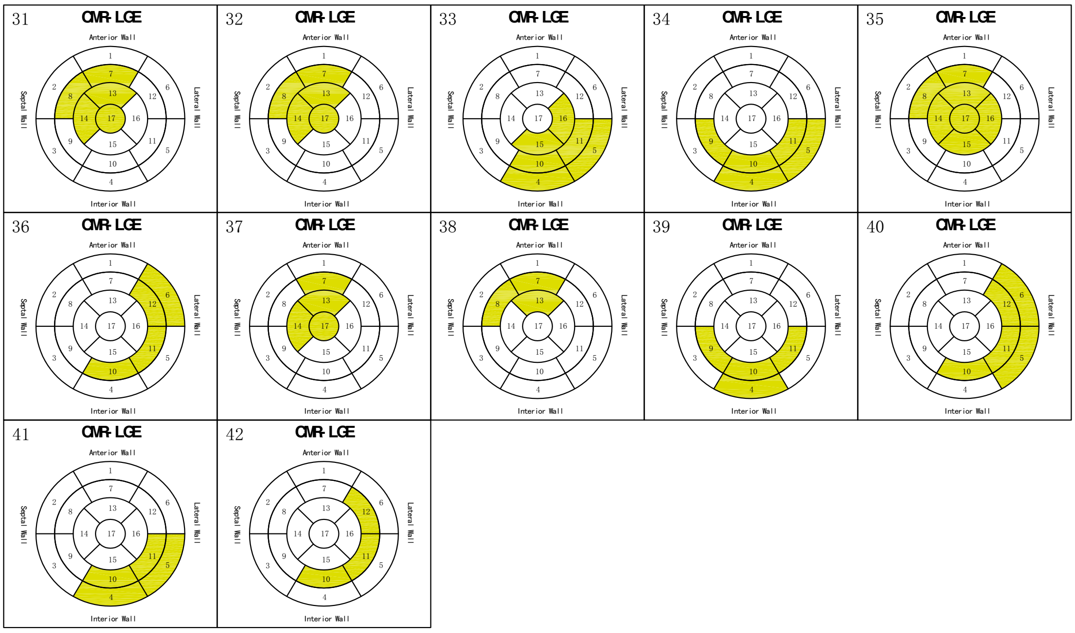

Supplemental Figure 1. Bull's Eye representation of segments in which visual analysis of CMR-LGE detected scar tissue.

Supplemental Figure 2.

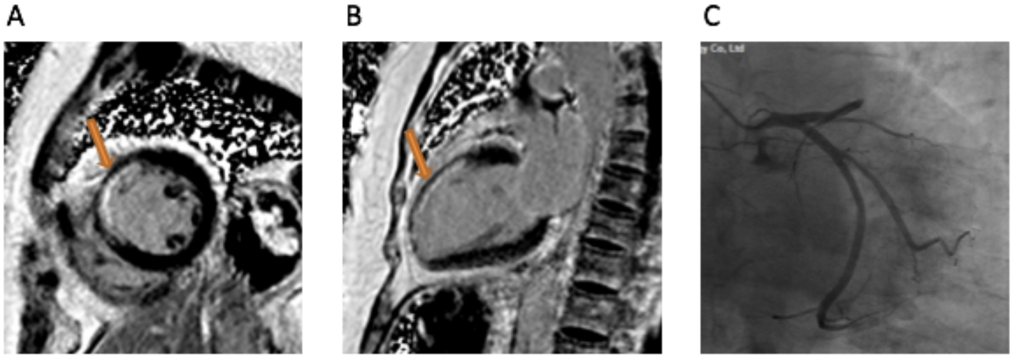

Supplemental Figure 2-1. Myocardial fibrosis in a STEMI patient lead by anterior descending coronary artery occlusion.

STEMI: ST-segment elevation myocardial infarction. Arrows indicate the scarred myocardium. Anterior wall of left ventricle in a short axis view imaged with CMR-LGE (A). Anterior wall of left ventricle in a 2-chamber view imaged with CMR-LGE (B). Coronary angiography (C) revealed occlusion of the anterior descending coronary artery.

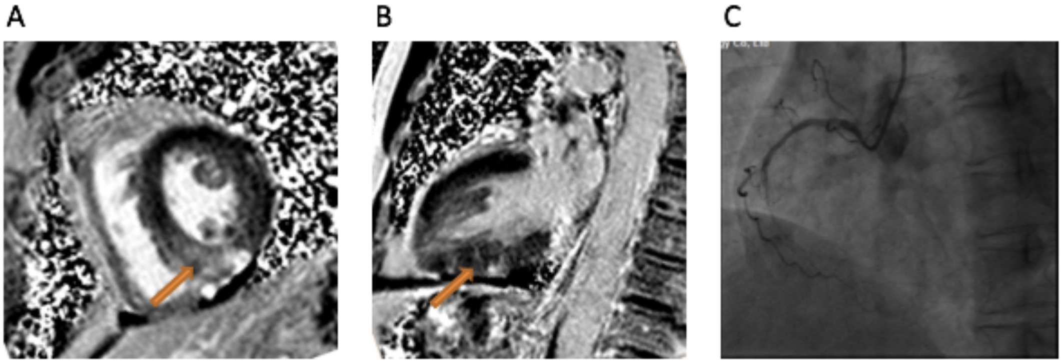

Supplemental Figure 2-2. Myocardial fibrosis in a STEMI patient lead by right coronary artery occlusion.

STEMI: ST-segment elevation myocardial infarction. Arrows indicate the scarred myocardium. Inferior wall of left ventricle in a 2-chamber view imaged with CMR-LGE (A). Posterior interventricular septum and inferior wall of left ventricle in a short axis view imaged with CMR-LGE (B). Coronary angiography (C) revealed occlusion of right coronary artery.

Supplemental Figure 3.

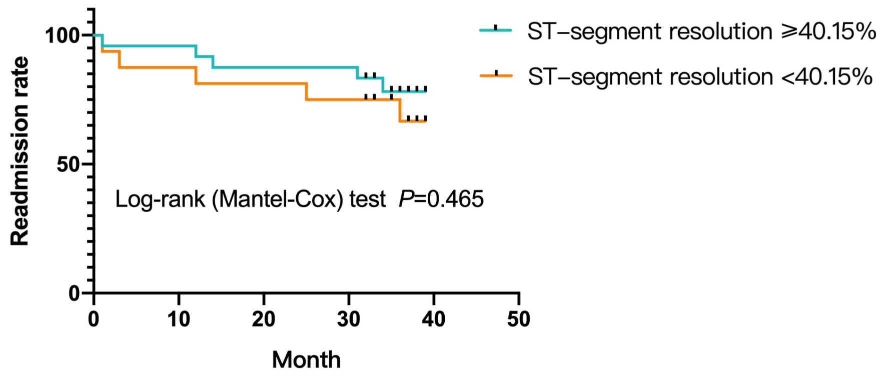

Supplemental Figure 3-1. The readmission rate 40 months after PCI were followed up in both groups.

The readmission rate of ST-segment resolution<40.15% was higher than that of ST-segment resolution≥40.15%, but there was no statistically different rate of readmission between the two groups, due to the study was not powered to demonstrate such a difference with the small sample size.

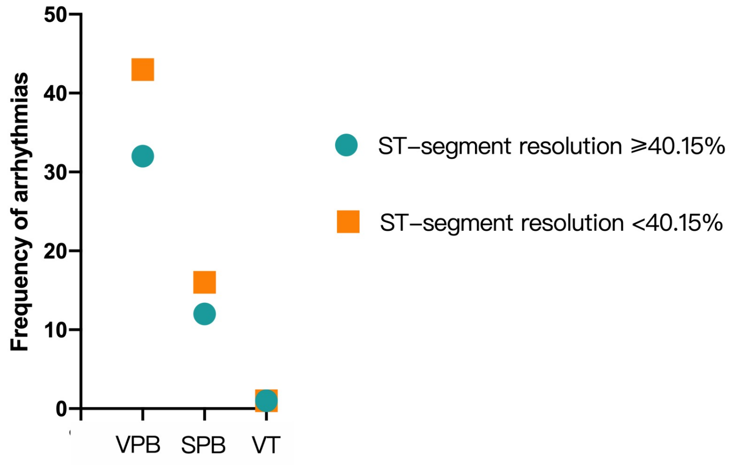

Supplemental Figure 3-2. The incidence of arrhythmias 40 months after PCI were followed up in both groups.

VPB: ventricular premature beats; SPB: supraventricular premature beats; VT: ventricular tachycardia. The incidence of arrhythmias of ST-segment resolution<40.15% was higher than that of ST-segment resolution≥40.15%, but there was no statistically different rate of the incidence of arrhythmias between the two groups, due to the study was not powered to demonstrate such a difference with the small sample size.
